# A survey of visual function in an Austrian population of school-age children with reading and writing difficulties

**DOI:** 10.1186/1471-2415-10-16

**Published:** 2010-05-25

**Authors:** Wolfgang Dusek, Barbara K Pierscionek, Julie F McClelland

**Affiliations:** 1 Vision Science Research Group, School of Biomedical Sciences, University of Ulster, Coleraine, Co. Londonderry BT52 1SA, UK

## Abstract

**Background:**

To describe and compare visual function measures of two groups of school age children (6-14 years of age) attending a specialist eyecare practice in Austria; one group referred to the practice from educational assessment centres diagnosed with reading and writing difficulties and the other, a clinical age-matched control group.

**Methods:**

Retrospective clinical data from one group of subjects with reading difficulties (n = 825) and a clinical control group of subjects (n = 328) were examined.

Statistical analysis was performed to determine whether any differences existed between visual function measures from each group (refractive error, visual acuity, binocular status, accommodative function and reading speed and accuracy).

**Results:**

Statistical analysis using one way ANOVA demonstrated no differences between the two groups in terms of refractive error and the size or direction of heterophoria at distance (p > 0.05). Using predominately one way ANOVA and chi-square analyses, those subjects in the referred group were statistically more likely to have poorer distance visual acuity, an exophoric deviation at near, a lower amplitude of accommodation, reduced accommodative facility, reduced vergence facility, a reduced near point of convergence, a lower AC/A ratio and a slower reading speed than those in the clinical control group (p < 0.05).

**Conclusions:**

This study highlights the high proportions of visual function anomalies in a group of children with reading difficulties in an Austrian population. It confirms the importance of a full assessment of binocular visual status in order to detect and remedy these deficits in order to prevent the visual problems continuing to impact upon educational development.

## Background

It is widely accepted that early detection and treatment of uncorrected refractive errors, binocular visual anomalies and/or amblyopia will reduce the risk of long-term visual problems. Recent studies have demonstrated that children with visual difficulties may be at an educational disadvantage to their visually normal peers with regard to educational attainment [1,2]. In addition, children with visual impairment are a greater risk of developmental setback in terms of sensorimotor understanding (non-verbal cognition), verbal comprehension, expressive language, social development and behavioural status [3]. Children with specific learning difficulties (e.g. dyslexia) and reduced intellectual ability are more likely to experience anomalies of visual function than their peers [4]. However, clinical experience suggests that a large number of children without specific learning difficulties or reduced intellectual ability also may experience problems with reading and writing. In this particular group of neurologically normal children it is likely that these visual difficulties may go unnoticed unless a comprehensive visual status assessment is performed. Reading difficulties are commonly associated with disorders of visual function, including binocular vision anomalies [5], and uncorrected refractive errors and may be easily detected and remedied following a full visual assessment. Reading difficulties may also result from perceptual visual disorders, which may be described as visual stress, visual discomfort or Meares-Irlen syndrome [6-8].

Whilst there is a large body of data detailing visual, reading and writing difficulties in children with reduced intellectual ability or specific learning difficulties, there is little data in the literature concerning the visual status of neurologically normal children with reading and writing difficulties, especially in a European population. Several studies that have been conducted have examined only one aspect of visual function (e.g. accommodative amplitude) and have not considered the range of measurements required for a functional assessment of vision [9-12].

Children in Vienna with difficulties in reading and writing are routinely referred by their teacher or parent to educational institutions for an academic assessment. Standardised assessments are performed by educational psychologists to help to determine the cause of the child's difficulties. An assessment will be used to establish whether the child has any specific learning difficulties or a reduced level of intelligence (low IQ). In Vienna, these assessments are carried out in one of three educational institutes (Holistic Institut, Förderpädagogisches Zentrum and Gesundheits Zentrum). A proportion of these children are found to have no specific learning difficulties and a normal level of intellectual ability despite their reading and writing difficulties. These particular children are routinely referred to optometric practitioners for a full assessment of visual status.

The present study aims to describe and compare the visual status of two groups of children attending a specialist Optometry practice in Vienna, Austria: one group referred to the practice from educational assessment centres diagnosed with reading and writing difficulties and the other, a clinical age-matched control group. The present study investigates the prevalence of refractive errors and binocular visual anomalies as well as reading ability in the two populations.

## Methods

### Subjects

Retrospective data from two separate groups of subjects has been examined for the present study. One group of subjects with reading and writing difficulties (referred group n = 825) were referred for an optometric assessment. These subjects were referred from three educational institutes in Vienna, Austria diagnosed with difficulties in reading and writing that could not be attributed to a learning difficulty. Reading difficulties were the primary concern of the children and their parents and no subjects reported writing difficulties in the absence of reading difficulties. All subjects had been assessed by an educational psychologist and had an IQ (intelligence quotient) over 70.

Data from a clinical control group of subjects (control group n = 328) were examined for comparison. Subjects in the clinical control group were young patients who attended the optometric practice for a routine eye examination, were not referred by an educational assessment centre and did not have difficulties with reading. All subjects and parents of the subjects in the clinical control group were asked specifically whether they had any difficulties with reading at home or in school. Subjects in the clinical control group who reported difficulties with reading were referred to an educational institute for an assessment of learning and were excluded from the study (n = 10).

Table 1 details the age and gender distribution of the two subject groups.

**Table 1:** Subject profile.

	Control Group	Referred group
**Number (n)**	328	825
**Mean age (years)**	9.34 ± 2.23	9.66 ± 1.84
**Age range (years)**	6-14	6-14
**Male/female**	193 males 135 females	531 males 294 females

Number, mean age, age range and gender distribution of subjects in the control group and the referred group.

Subjects with ocular pathology (e.g. cataract, glaucoma, strabismus) or learning difficulties were excluded from the study (n = 8) and referred for ophthalmological investigation.

All children in the present study in both groups were attending mainstream schools. Ethical approval for the study was obtained from the University of Ulster Research Ethics Committee and the study adhered to the tenents of the Declaration of Helsinki. Informed consent was obtained from the parents to allow inclusion of their child's data in the study.

### Procedure

The following measurements were obtained from each participant's clinical records. All methods are routine procedures commonly performed in Optometric practice and were carried out by Mr Dusek, an Optometrist with many years of clinical experience in testing children.

#### Questionnaire

The optometric clinical records of children referred to WD's Optometry practice were retrospectively examined. All subjects were given a questionnaire which contained standard clinical questions (Additional file 1) to ascertain whether they were experiencing any specific visual/ocular problems. These questions were directed to the child in the presence of the parent. All questions were asked in a standard order.

#### Visual Acuity

Distance visual acuity was assessed with a commonly used European optometric test chart, the Polatest at a distance of 5 m. The Zeiss Polatest is back illuminated and uses full contrast letters recorded in Snellen acuity decimal form (ISO 8597) [13-15]. Subjects were asked to name or match letters on the chart. A standard procedure was applied. If a clinically significant refractive error was found (≥ +1.00D hyperopia, ≤ -0.50D myopia, ≤ -1.00DC astigmatism or ≥ 1.00D anisometropa), visual acuity was assessed on a separate occasion with the full spectacle prescription in place.

#### Refractive Error

Refractive error was assessed using standard distant static retinoscopy. [16]

#### Ocular Posture

A standard cover-uncover test revealed the presence of heterotropias and heterophorias at distance and near (5 m and 40 cm). The subject was asked to maintain fixation on an acuity appropriate target on the Polatest while each eye was covered and uncovered. The subject was allowed to fixate the target for three seconds before the eye was covered and uncovered. The presence and direction of movement was noted. A prism cover test was employed to assess the magnitude of the deviations present [17].

#### Ocular motility

The subject was asked to keep his/her head as still as possible and to follow a pen torch with their eyes. They were asked to report any diplopia or discomfort. Any restrictions were noted.

### Accommodation

The amplitude of accommodation was measured monocularly using the push-up test [18-20].

Accommodative facility was assessed in cycles per degree both monocularly and binocularly using flipper lenses (+2.00/-2.00). This was repeated for one minute and the number of cycles was noted [21].

Monocular Estimation Method (MEM) retinoscopy was used to assess the accommodative response to a target at a specific distance. The distance refractive error was fully corrected and a near target attached to the retinoscope at a distance of 40 cm. The subject was encouraged to read the text aloud while the retinoscopy reflex was observed. If a 'with' movement was detected, plus lenses were added in 0.25 steps until neutrality was achieved and if an 'against' movement' was observed, minus lenses were added in 0.25 steps until neutrality was achieved [22].

AC/A ratio was assessed by measuring the near phoria at 40 cm using the alternating cover test and prism bar. This was then repeated using -2.00 lenses while the subject maintained fixation on the target at 40 cm. AC/A ratios were classified as follows; low <2:1, normal 2:1 to 5:1 and high >5:1.

### Convergence

Near point of convergence (NPC) was assessed using a standard procedure. The subject was asked to fixate on the light of a pen torch while it was moved closer to the subject. The subject was asked to report the point when diplopia was first noticed. The clinician also objectively observed the point at which the subject lost fixation, when one eye deviated. The points at which the subject and the observer noticed a loss of fixation were noted [20,23,24].

Vergence facility was assessed in cycles per degree using flip prism 3ΔBI/12ΔBO, giving information about the condition and the speed of the convergence system. Subjects were asked to fixate a small target on the Gulden stick at 40 cm and asked to try to keep the target single and clear. Prism (3Δ (base in (BI)) was introduced first and the child asked to report when it became single. When the target was single and clear the 12Δ (base out) BO was introduced. When the child reported that the target was clear the prism was switched back to the 3ΔBI. This was repeated for one minute and the number of cycles was noted [25].

#### The accommodative convergence system of the eyes

The AC/A ratio was assessed by measuring the near phoria at 40 cm using the alternating cover test and prism bar. This was then repeated using -2.00D lenses in front of the eyes while the subject maintained fixation on the target at 40 cm. The AC/A ratio was calculated as the difference between the measured phoria with and without the -2.00D lenses, divided by two [26].

#### Reading speed

Reading speed was assessed using a standard Austrian test known as The Salzburg Reading Test [27]. Age appropriate material suitable for that particular subject was selected and the test was conducted in a quiet room. Each child was asked to start reading the section of text suitable for his/her age group. The time taken to complete the task was measured with a stopwatch, the number of incorrect words read noted and a score calculated [27]. The subjects were asked to read a selection of both simple words (e.g. tree, house, car) and fantasy words (nonsense words such as talire and holotu).

## Results

Retrospective clinical data from 1153 subjects were examined.

### Questionnaire

All subjects in both groups provided an answer for all 14 questions. Statistical analysis (one way ANOVA) demonstrated that subjects in the referred group were more likely to complain of; burning or stinging eyes, tiredness after reading, eye strain when looking at a near target, blurred vision at near, blurred vision at distance and diplopia (p < 0.01) (Cohen's d ranged from 0.16 to 0.95). There was no statistically significant difference between the two groups for the other twelve questions (p > 0.05).

### Refractive error

Statistical analysis (one way ANOVA) demonstrated no significant differences in refractive error (sphere and cylinder) between the right and left eyes in both groups (p > 0.05). Therefore, for all analyses using refractive error, only the right eye was considered. Table 2 details the mean, standard deviation and range of spherical and cylindrical refractive errors in both groups.

**Table 2:** Refractive error.

	Sphere (D)		Cylinder (D)	
	Control Group	Referred Group	Control Group	Referred Group
**Mean**	+0.38	+0.30	-0.18	-0.13
**Standard deviation**	± 1.56	± 1.17	± 0.56	± 0.39
**Range**	-8.00 to +8.00	-5.75 to +9.50	-3.00 to 0.00	-3.50 to 0.00

Mean, standard deviation and range of spherical and cylindrical refractive errors in the control group and the referred group.

Statistical analysis using one way analysis of variance (ANOVA) demonstrated no significant difference between the spherical or cylindrical refractive error between the two groups (p = 0.307).

Clinically significant anisometropia was present in 10 subjects in the referred group and 18 subjects in the control group. Results showed no significant difference between the anisometropic error (≥ 1.00D) between the two groups (ANOVA p = 0.121).

### Visual Acuity (VA)

**Figure 1:**
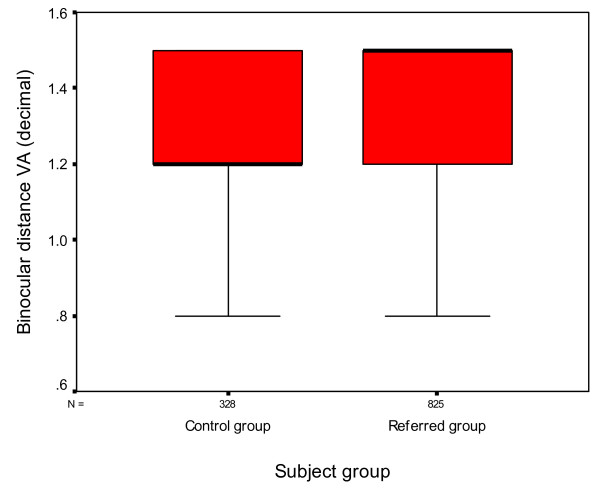
**Box and whisker plots of distance binocular visual acuity for each group**. The median VA is represented by the thick black lines inside the box. The upper and lower edges of the box represent the upper and lower quartiles of the data. The whiskers represent the highest and lowest values that are not outliers or extreme values.

Statistical analysis (one way analysis of variance ANOVA) demonstrated a significant difference in distance visual acuity (binocular) between the two groups with those subjects in the control group demonstrating poorer visual acuity than those in the referred group (p < 0.01 Cohen's d = 0.42) (Figure 1).

### Ocular Posture

**Figure 2:**
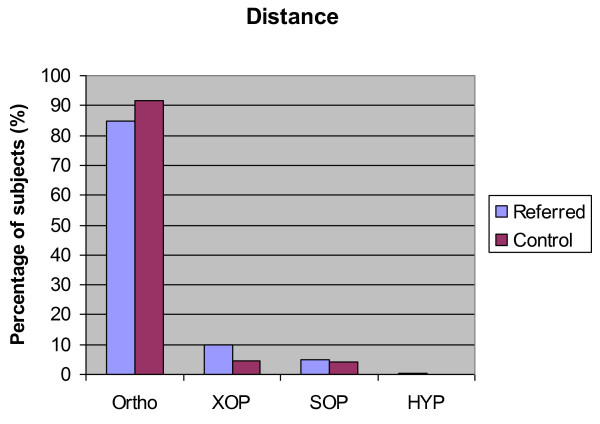
**Distance ocular posture status for both groups**. Histogram detailing the percentage of subjects in both the control group and the referred group with orthophoria (Ortho), exophoria (XOP), esophoria (SOP) and hyperphoria (HYP) at distance (5 m).

**Figure 3:**
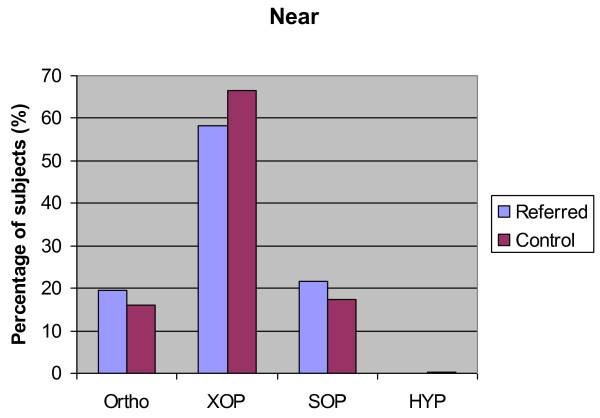
**Near ocular posture status for both groups**. Distance ocular posture status for both groups. Histogram detailing the percentage of subjects in both the control group and the referred group with orthophoria (Ortho), exophoria (XOP), esophoria (SOP) and hyperphoria (HYP) at near (40 cm).

Figures 2 and 3 show the results of the cover test in both groups.

Statistical analysis using one way ANOVA demonstrated no significant difference between the size or direction (esophoric or exophoric) of the phoria at distance between the control group and the referred group (p = 0.275 and p = 0.484 respectively) (Table 3).

**Table 3:** Ocular posture status.

	Exophoria				Esophoria			
	Distance		Near		Distance		Near	
	Control	Referred	Control	Referred	Control	Referred	Control	Referred
**Number (n)**	15	81	217	481	13	40	57	179
**Mean prism**	0.87	1.25	3.53	5.41	0.69	1.69	4.33	4.95
**Standard deviation**	2.1	2.85	3.27	4.68	1.7	3.76	3.63	4.49
**Percentage of subjects**	4.6	9.8	66.5	58.3	4	4.8	17.4	21.7

Number and percentage of subjects exhibiting exophoria and esophoria at distance (5 m) and near (40 cm) for the control group and the referred group. Table 3 also describes the mean size and standard deviation of the heterophoria in each group.

Analysis demonstrated no statistically significant difference between the size of esophoric deviations measured using the near cover test between the two groups (p = 0.48). Results showed a difference between the size of the exophoric deviation between the two groups with subjects in the referred groups tending to have a larger deviation (one way ANOVA p < 0.005 Cohen's d = 0.15).

A significant heterophoria (greater than 1/2 prism dioptre vertically and 2 prism dioptres horizontally) was found in 2 of 328 (0.6%) in the control group and 9 of 825 (1.1%) in the referred group. This difference was not statistically significant (p = 0.738).

### Accommodation

#### Amplitude

**Figure 4:**
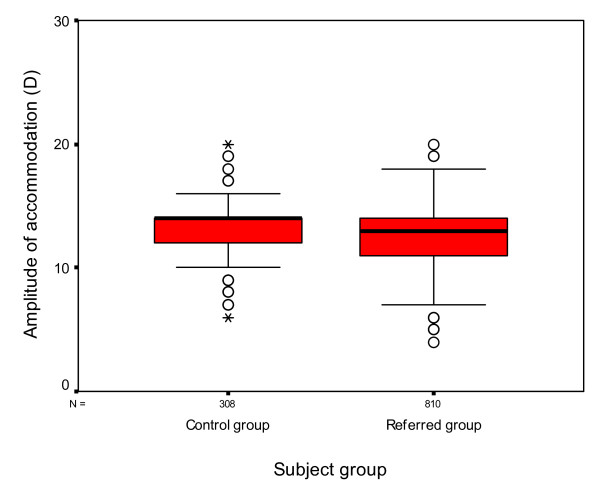
**Box and whisker plot of amplitude of accommodation for both groups**. The median amplitude of accommodation is represented by the thick black lines inside the box. The upper and lower edges of the box represent the upper and lower quartiles of the data. The whiskers represent the highest and lowest values that are not outliers or extreme values. The open circles represent the outlying data points. An asterisk marks extreme cases.

The amplitude of accommodation was assessed successfully with 308 subjects in the control group and 810 subjects in the referred group. Those subjects for whom amplitude of accommodation could not be assessed tended to be those subjects who were less co-operative. The mean amplitude of accommodation was 13.29D ± 2.05 in the control group and 12.54D ± 2.60 in the referred group. Amplitude of accommodation ranged from 6-20D in the control group and 4-20D in the referred group. Statistical analysis using one way ANOVA demonstrated a significant difference between the two groups (p < 0.001 Cohen's d = 0.32) with subjects in the referred group tending to have lower amplitudes of accommodation than those in the control group (Figure 4).

#### Accommodative facility

Accommodative facility was assessed successfully with 275 subjects in the control group and 783 subjects in the referred group. Statistical analysis (one way ANOVA) demonstrated a significant difference between the groups on the binocular accommodative facility test (p < 0.01 Cohen's d = 0.68). The mean measurement in the referred group was 6.51 ± 3.83 cycles per minute and in the control group 9.00 ± 3.46 cycles per minute.

Statistical analysis of results (one way ANOVA) obtained from the monocular accommodative facility test demonstrated a significant difference between the groups (p < 0.001 Cohen's d = 0.28). The mean measurement in the referred group was 12.00 ± 3.28 cycles per minute and 12.76 ± 1.93 cycles per minute in the control group.

Statistical analysis demonstrated that subjects in the referred group (4.4%) were more likely to have accommodative insufficiency than those in the control group (0.6%) (Chi-Square p = 0.001 Cramer's V = 0.13). Accommodative excess was only found in 2 out of 825 in the referred group (0.2%).

#### Vergence facility

Vergence facility was assessed with 274 subjects in the control group and 776 subjects in the referred group. One way ANOVA demonstrated a statistically significant difference between results obtained from the two groups (p < 0.001 Cohen's d = 0.81), (mean 7.43 ± 4.53 cycles per minute in the referred and mean 10.68 ± 3.42 cycles per minute in the control group).

#### AC/A ratio

**Figure 5:**
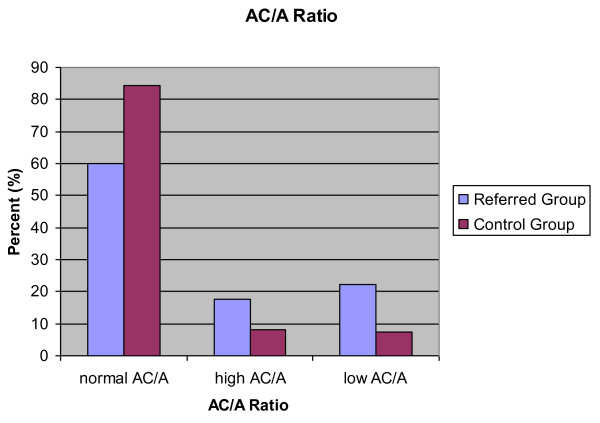
**AC/A ratio**. Percentage of subjects in both the control group and referred group classified as having a normal (2:1 to 5:1), high (>5:1) or low (<2:1) AC/A ratio.

AC/A ratio was assessed for 328 subjects in the control group and 825 subjects in the referred group. A normal AC/A ratio was demonstrated by 277 subjects in the control group (84.5%) and 496 subjects in the referred group (60.1%). A low AC/A ratio was demonstrated in 24 subjects in the control group (7.3%) and 183 subjects in the referred group (22.2%). A high AC/A ratio was demonstrated by 27 subjects in the control group (8.2%) and 146 subjects in the referred group (17.7%) (Figure 5).

### Subjects with normal AC/A ratio

**Figure 6:**
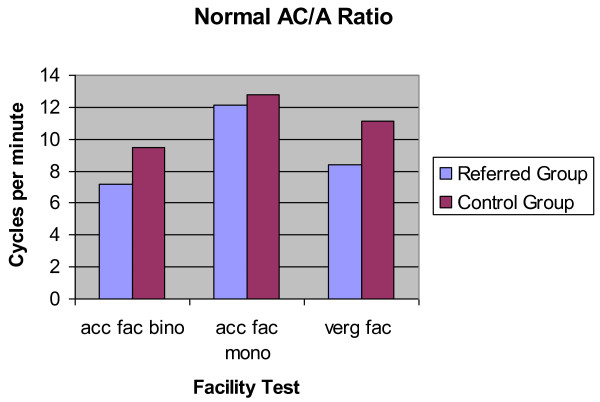
**Accommodative and vergence facility**. Accommodative and vergence facility test results (in cycles per minute) obtained from subjects with a normal AC/A ratio in both the control group and the referred group. The first column represents the binocular accommodative facility test results (acc fac bino), the second column represents the monocular accommodative facility test results (acc fac mono) and the third column represents the vergence facility test results (verg fac).

Considering only those subjects with normal AC/A ratios a statistically significant difference is found between the binocular and monocular accommodative facility tests in the two groups (p < 0.001 Cohen's d = 0.64 and p < 0.001 Cohen's d = 0.23 respectively). Subjects in the referred group (binocular mean 7.15 ± 3.89 cycles per minute, monocular mean 12.16 ± 3.18 cycles per minute) were more likely to have a lower accommodative facility measurement than those in the control group (binocular mean 9.49 ± 3.30 cycles per minute, monocular mean 12.76 ± 1.98 cycles per minute) (Figure 6).

The vergence facility test show similar results. Vergence facility measurements are statistically significantly lower in the referred group (mean 8.38+4.55 cycles per minute) than the control group (mean 11.15 ± 3.07 cycles per minute) (p < 0.001 Cohen's d = 0.71).

A dysfunction of the accommodative vergence system was found in 49 of 328 (14.9%) in the control group and in 280 of 825 (33.9%) in the referred group (Figure 6). Statistical analysis demonstrated a significant difference between these two group (Chi-Square p < 0.001 Cramer's V = 0.19).

### Convergence

Near point of convergence (NPC) was assessed successfully with 324 subjects in the control group and 801 subjects in the referred group. The mean NPC was 3.41 ± 4.62 cm in the control group and 4.74 ± 5.59 cm in the referred group. Statistical analysis demonstrated a significant difference between the two groups (p < 0.001 (Cohen's d = 0.26).

Convergence excess was diagnosed in 27 out of 328 (8.2%) in the control group and 140 out of 825 (17%) in the referred group. Statistical analysis demonstrated a significant difference between these two groups (Chi-Square p < 0.001 Cramer's V = 0.11).

Convergence Insufficiency was found in 17 of 328 (5.2%) in the control group and in 150 of 825 (18.2%) in the referred group. Statistical analysis demonstrated a significant difference between these two groups (Chi-Square p < 0.001 Cramer's V = 0.17).

### Reading Speed

Reading speed was assessed successfully with 764 subjects in total (285 in the control group and 479 in the referred group).

**Figure 7:**
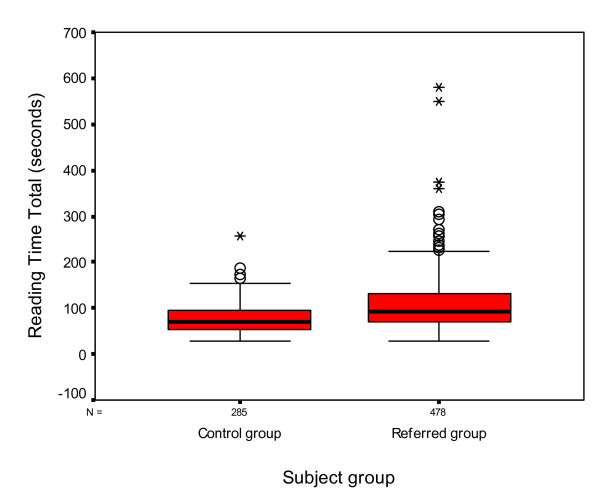
**Box and whisker plot of reading time with Salzburg Reading Test for both groups**. The median total reading time is represented by the thick black lines inside the box. The upper and lower edges of the box represent the upper and lower quartiles of the data. The whiskers represent the highest and lowest values that are not outliers or extreme values. The open circles represent the outlying data points. An asterisk marks extreme cases.

**Figure 8:**
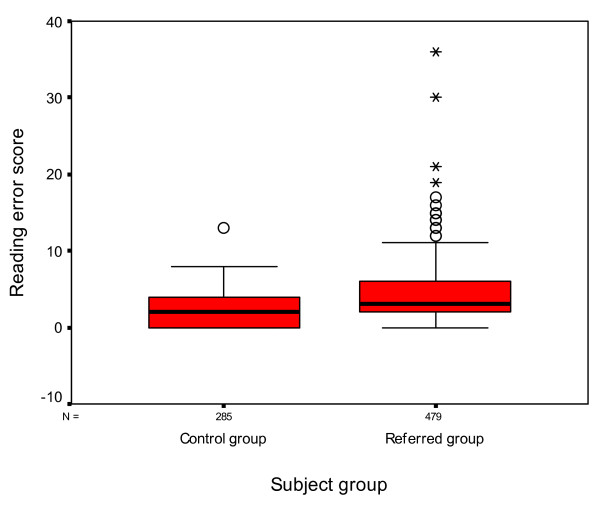
**Box and whisker plot of reading error scores with the Salzburg Reading Test**. The median reading error score is represented by the thick black lines inside the box. The upper and lower edges of the box represent the upper and lower quartiles of the data. The whiskers represent the highest and lowest values that are not outliers or extreme values. The open circles represent the outlying data points. An asterisk marks extreme cases.

The reading speed total is the sum of the time taken to read both the simple words and the fantasy words (48 words in total). The reading error total is the number of errors made in the simple words and fantasy words. Statistical analysis using an one way ANOVA demonstrated a statistically significant difference between the results obtained from each group with those subjects in the referred group showing an increased reading time (referred group mean time 108.64 ± 61.7 seconds, control group mean 75.98 ± 32.78) (p < 0.001 Cohen's d = 0.66) and a larger number of errors (referred group mean error score 4.28 ± 3.77, control group mean error score 2.29 ± 2.18) (p < 0.001 Cohen's d = 0.65) (Figures 7 and 8).

### Ranking

The Salzburg Reading Test also compares the subject's individual score with age-matched control data and states the percentage of children of the same age that read more slowly than the particular subject being tested. This is known as the ranking total.

**Figure 9:**
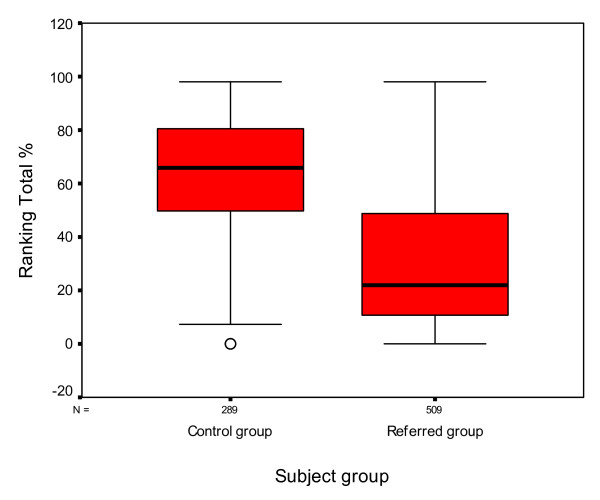
**Box and whisker plot of ranking results for the Salzburg Reading Test for both groups**. The median ranking is represented by the thick black lines inside the box. The upper and lower edges of the box represent the upper and lower quartiles of the data. The whiskers represent the highest and lowest values that are not outliers or extreme values. The open circles represent the outlying data points.

Figure 9 shows that children in the control group had a higher ranking total score on the Salzburg reading test than the referred group.

No significant visual function anomalies are apparent in 186 of 328 (56.7%) in the control group and 127 of 825 (15.4%) in the referred group.

### Visual function

In both groups, subjects with lower amplitudes of accommodation are more likely to have a slower reading time (regression analysis r = 0.094 and p < 0.01), reduced accommodative response (regression analysis r = 0.31 and p < 0.01) and reduced accommodative binocular facility (regression analysis r = 0.21) and p < 0.01). There are no statistically significant associations between all other measures of visual function (p > 0.05).

## Discussion

Due to the nature of the study it was not possible to mask the researcher (WD) to the subject category (control or referred group). However, in order to minimise the potential for bias, all testing procedures and conditions were standardised for each subject.

Questionnaires have been used widely to investigate visual symptoms in children and appear to be an appropriate tool to determine the presence of asthenopic symptoms [28-31]. Analysis of the questionnaire results demonstrated that those children referred for an assessment were more likely to complain of fatigue and various asthenopic symptoms, however in addition to highlighting reading difficulties this questionnaire may simply be indicating the presence of optometric anomalies. The authors suggest that further work investigating the presence of visual stress, visual discomfort or Meares-Irlen syndrome is indicated in this particular group of subjects.

Results demonstrated that those subjects in the referred group were statistically more likely to have binocular vision anomalies: exophoria, reduced convergence, lower amplitudes of accommodation and a lower AC/A ratio. The referred group also demonstrated a reduced reading speed compared to controls.

Although, it is well accepted that children with learning difficulties, and in particular those associated with dyslexia, are more likely to have visual problems, there is a group of children whose visual problems may be overlooked because they attend mainstream education and are classified as intellectually and visually 'normal'. There are no obvious signs of any anomalies, visual acuity is relatively good and symptomatically, these children appear to differ little from children with no reading/writing difficulties. Yet a number of symptoms (burning/stinging sensations, asthenopia, eyestrain, blurred vision are near and distance, and diplopia) were significantly more frequently reported in children with reading difficulties compared with controls. It is worth noting that, in a previous study on Swedish school children (between 5.8 and 9.8 years of age), none of whom were referred for reading problems, over a third reported symptoms with near work at the first examination and this increased to over 40% at the second examination [9]. Notably, children younger that 7.5 years, did not report symptoms. It was not possible to determine whether this was because of a true absence of symptoms or because children younger than this age did not understand the questions.

The importance of binocular vision status on scholastic effort cannot be underestimated. Anomalies of binocular vision, including heterophorias, disorders of vergence and accommodation, if left untreated, can lead to difficulties in reading and writing that will increase with each year in school as educational demands grow. The value of healthy binocular vision extends beyond scholastic effort and achievement. Poor vergence and/or accommodative capacity will impact on sporting performance, balance and coordination and can lead to a depletion in self-confidence. In addition, these anomalies of vision may be compounded by underlying visual perceptuial difficulties such as visual stress (visual discomfort or Meares-Irlen syndrome) [7.8].

In addition to ignoring binocular vision problems, the other potential obstacle to educational and associated development in these children, is that of being misdisagnosed as dyslexics. The subsequent treatments offered would be at best inappropriate and at worst, could lead to further detriment in development.

It should be noted that whilst information on other health issues was not available for these children, the Austrian system requires regular health checks for all school age children and any serious conditions would have resulted in referral to other specialists and these would have been made known to subsequent practitioners. Similarly, although intelligence quotients and other measures of intellectual ability were not measured by the authors, children in mainstream education and those referred from the Institutes, were deemed to have normal or above normal intelligence levels, as reported by the referring educational psychologist.

As it is well accepted that vergence and accommodative problems can be comparatively easily treated, the importance with regard to developmental process and education may be underestimated [32]. Some studies have considered aspects of binocular vision but have not looked at all relevant parameters. A study, from Spain considered only accommodative function of 87 children with reading problems and 32 controls [10]. The findings support those of this study: that reading problems in children who are intelligent enough to be educated in mainstream schools and were not dyslexic, are associated with a reduced amplitude of accommodation. No account, however, was taken of other binocular variables. A study on 76 Polish schoolchildren, in the second year of school (average age 8.75 years) also considered only the contribution of accommodative infacility on scholastic and sporting performance [11]. No correlation between the latter two variables and accommodative capacity was found but it must be noted that this study was not conducted on children with reading difficulties.

An earlier study from Denmark [12] examined a range of binocular functions (visual acuity, phorias, tropias, fusional amplitudes and stereopsis) in addition to visual acuity and refraction on 41 primary school children with poor reading ability and 200 good readers. No statistically significant difference was found between the two groups. There were no measurements of accommodative amplitude or facility.

## Conclusions

There are insufficient studies on the incidence and prevalence of binocular vision problems in school children and how this may equate with educational and other achievements. This study is the first of its kind in Austria. Further studies from other European countries are required in order to assess the demographic extent of the problem in Europe, to monitor changes in time and to evaluate the outcome of treatments. Studies from beyond the European region are also needed. The value of a multi-disciplinary team approach with eyecare practitioners, vision specialists, educationalists and psychologists cannot be underestimated.

## Competing interests

The authors declare that they have no competing interests.

## Authors' contributions

All authors read and approved the final version of the manuscript. WD designed and carried out data collection and analysis. JM participated in the design of the study, data analysis and preparation of the manuscript. BP participated in the design of the study, data analysis and preparation of the manuscript.

## Pre-publication history

The pre-publication history for this paper can be accessed here:

http://www.biomedcentral.com/1471-2415/10/16/prepub

## Supplementary Material

Additional file 1 **Appendix - Questionnaire**. Ocular history and symptoms questionnaire Click here for file
