# Supplementary material for: A survey of visual function in an Austrian population of school-age children with reading and writing difficulties
Source: BMC Ophthalmol. 2010 May 25;10:16. doi: 10.1186/1471-2415-10-16 (PMC2887790; doi:10.1186/1471-2415-10-16)
Supplement: Additional file 1 — Appendix - Questionnaire. Ocular history and symptoms questionnaire [file 1471-2415-10-16-S1.DOC]

Dear Editor,

**RE: MS: 2133312039323961**

**Title:** A survey of visual function in an Austrian population of school-age children with reading and writing difficulties.

**Authors:** Wolfgang Dusek, Barbara K Pierscionek and Julie F McClelland

Please find attached an amended version of the manuscript with changes highlighted in yellow and a point-by point response to the reviewers’ comments (below). Please also see below for a response to the Editor’s comments. We are pleased that the reviewers agree that the manuscript will be a valuable contribution to the literature in this area.

We are very grateful to the reviewers for their positive and helpful suggestions and we feel that the quality of the manuscript has been significantly improved as a result.

We look forward to seeing our manuscript in your journal.

Yours sincerely,

Julie McClelland

Wolfgang Dusek

Barbara Pierscionek

**Response to Editor’s comments**

Please add more information to the background section of your abstract to place your study in context.

**The background section of the abstract has been amended as suggested.**

Please move the 'competing interests', 'author's contributions' and 'acknowledgements' sections of your manuscript so that they are located after your conclusions section, but before your reference list.

**The 'competing interests', 'author's contributions' and 'acknowledgements' sections of the manuscript have now been moved so that they are located after the conclusions section, but before the reference list.**

We would be grateful if you could address the comments in a revised manuscript and provide a cover letter giving a point-by-point response to the concerns.

**A point-by-point response has been provided below.**

Please also highlight (with 'tracked changes'/coloured/underlines/highlighted text) all changes made when revising the manuscript to make it easier for the Editors to give you a prompt decision on your manuscript.

**The changes have been highlighted in yellow.**

**REVIEWER 1**

**Reviewer's report**

**Title:** A survey of visual function in an Austrian population of school-age children

with reading and writing difficulties

**Version:** 3 **Date:** 23 December 2009

**Reviewer:** Elizabeth G Conlon

**Reviewer's report:**

This is an interesting paper with an impressive sample size. This paper reports that many children who have some difficulties reading also have visual difficulties in a number of optometric functions. It concludes importantly that these types of difficulties can contribute to the educational difficulties in reading and writing experienced by these children.

While I would like to see this work published there are a number of issues that

need to be addressed to streamline and strengthen the paper. These are all

compulsory revisions for this manuscript.

I am writing this review from the perspective of a dyslexia researcher who

specialised in visual perceptual processing in reading and reading disability. As such I cannot directly comment on the methodology of the optometric tests.

**The authors are pleased that this reviewer feels that the manuscript draws important conclusions. We are very grateful to the review for her helpful comments regarding the manuscript and we feel that the suggested changes have improved the standard of the paper significantly. Please find below details of how each point has been fully addressed.**

1. A question that has produced major controversy in the area of visual processes in reading and dyslexia is the co-occurrence of the visual perceptual difficulty referred to as visual stress, visual discomfort, or Meares-Irlen Syndrome. Many of the types of difficulty found in the clinic sample are similar to the types of difficulties reported by individuals with that particular anomaly. This manuscript would be considerably strengthened by some discussion of this anomaly and how the optometric difficulties found in the sample may explain a number of the difficulties reported. This would increase the relevance of the findings to the wider research on visual processes in reading. Interestingly individuals with visual stress have a slow reading rate, but few phonological difficulties which separates these persons from others with dyslexia. (Some individuals do have both types of difficulty. A proportion of the visual perceptual difficulties may be partially or fully explained by undiagnosed optometric difficulties.)

**Information regarding visual stress has been added to both the introduction and discussion. The authors agree that this has strengthened the manuscript.**

Specific comments.

2. Methods/results section from abstract. Simply writing statistical analysis was conducted is not explanatory. Independent groups t-tests and chi-square

analyses were predominantly conducted.

**The types of statistical tests used have now been included in the abstract.**

3. Results in abstract. Lower reading speed, should be slower reading speed.

(discretionary)

**This has now been amended.**

Tables and Figures

4. The figures and Tables are very difficult to follow. While two group analyses have been reported, scatter diagrams with regression lines have been reported from SPSS printouts. These are commented on more fully when commenting on the results section of the manuscript.

**These comments have been addressed specifically in the results section.**

Introduction

5. In the background section, it would be helpful to expand the sorts of perceptual difficulties discussed. This is mentioned in the general comments above.

**The introduction has been amended to include information on visual stress and Meares-Irlen syndrome.**

6. P.4 third last line of introduction. “looks at” should read ‘investigates”

(discretionary)

**This has now been changed.**

7. Subjects. While it is reported in this section, that all children had an IQ over 70 in a later part of the paper it was reported that this was not assessed.

**Whilst the IQ was not directly assessed by the authors, it was reported by the referring educational psychologist. This has now been clarified in the manuscript.**

**This has been now amended to read, ‘Similarly, although intelligence quotients and other measures of intellectual ability were not measured by the authors, children in mainstream education and those referred from the Institutes, were deemed to have normal or above normal intelligence levels, as reported by the referring educational psychologist.**

8. Children with an IQ of 70 would have poorer reading skills than other children. Cut-off points for ability in most research on dyslexia is 85 or in many cases 90.

**This is the information that we had available from the referring educational psychologist. Unfortunately it was not possible to have more specific detail on the IQ levels of the children involved in the study. It should also be noted that IQ tests and their cut-off points may vary slightly depending on the language in which they are presented.**

9. Questionnaire. Many of these questions are similar to those asked of individuals when assessing them for visual stress – My question here is how well the children understood the different types of difficulty experienced? (Discretionary)

**Both questions were asked to each group in exactly the same manner. If the subject appeared to have any difficulty understanding the question this was rephrased and asked again with further explanation from the parent if necessary. The authors agree that some children may have difficulty understanding the questions asked however, questionnaires or ‘symptom surveys’ are widely used in optometric and specifically binocular vision research and have been shown to be valid and reliable (references 26 and 27).**

10. Reading speed. Salzburg reading test – this is not referenced.

**A reference has now been included.**

11. In the table that refers to the findings from the test, errors in simple words and fantasy words are reported. The authors should describe what simple and fantasy words are. For example, are fantasy words nonsense words? Are simple words phonologically regular words, or single syllable words?

**This has now been further explained and examples of both simple and fantasy words have been provided in the manuscript.**

12. Results. Parts of this were hard to follow. With a very large sample size and a far greater number of children in the clinical group, the standard deviation of the clinical group would be expected to be larger than the SD of the control group simply as a result of sample size.

Some of the results appear to produce large differences between these groups.

As all of the parametric analysis is two group analyses, the effect size Cohen’s d should be reported for each of the effects. When N is large, power is very high and negligible results sometimes produce statistically significant effects. The effect sizes would provide strong evidence of the strength of the different effects found. Cramers V or some other measure of effect size, for example an odds ratio could be presented to demonstrate the size of the effect for the chi-square analyses.

**Cohen’s d results have now been provided for each ANOVA to clarify effect size. Cramer’s V results have also been provided for all the chi-square analysis data.**

13. Why are t-tests reported on some occasions and one-Way ANOVA on others.

14. The two tests are similar (F = t2). It would be more systematic to use the same analyses.

**All data analysed using t-tests have now been reanalysed using ANOVA.**

15. The tables and figures that describe each of the different results were

very difficult to follow. There are a large number of figures. These are not all

referred to. On page 9 Figure 13 is referred to. I could not find a figure 13.

**The reference to the figure 13 has now been removed. The authors apologise for this oversight.**

16. The purpose of the scatter diagrams with the regression lines added is unclear. Regression analyses was not conducted across age groups.

In Figure 4, for example, in addition to the scatter diagram showing age groups, it is reported that the SD of the referred group was large. Inspection of the figure show that the referred group predominate at the two ends of the distribution, with some showing low and other high amplitude of accommodation. Do these children differ on other measures of optometric ability?

**Figure 4 has been replaced with a box plot to improve presentation of the data.**

**The children with lower amplitudes of accommodation are also more likely to have a slower reading time, reduced accommodative response and reduced accommodative binocular facility (p<0.01), however, there are no statistically significant associations with the other measures of visual function (p>0.05). This has now been stated in the manuscript.**

17. Some measure of variability, for example the standard error, standard deviation or the confidence interval would assist on the bar charts. Figures 7 to 9 show a great deal of variability in the referred group for the reading speed and reading error components of the data analysis.

**Figures 7, 8 and 9 have now been replaced with box and whisker plots. The authors believe that these plots better represent the range and variability of data collected.**

18. Do those children with the slowest reading rate also have the most severe optometric difficulties? The figures should be clarified and the number presented reduced.

**This information has now been included in the manuscript.**

19. What information does Figure 1 supply to the reader?

**Figure 1 has now been amended to better represent the data.**

20. In some cases means and standard deviations are reported in the text, tables

and then figures are also presented. These should be simplified and information concerning the description of each analysis reported once.

**Data has been removed from table 3; table 4 has been removed in order to simplify the results.**

21. Do the children in the referred group without visual anomalies have a similar reading performance to non-clinic children of similar age? Do children with visual anomalies in the control group, read more slowly than the other children in their age group without visual anomalies. These analyses would assist when clarifying the extent that the visual anomalies reported contribute to the poorer reading speed reported on the reading test. (This may be considered by the authors to be discretionary. I think that this would be interesting as it may provide some insight into the effects of the types of optometric difficulties reported on the measure of reading skill measured.)

**Yes, some visual anomalies, (especially those that involve near vision, such as amplitude of accommodation) are associated with a slower reading speed. This information has now been included in the manuscript.**

Discussion

22. While some differences were found between the referred and non-referred

groups on some items on the questionnaire, and the authors report that these

types of questionnaires are used frequently to obtain self reports of difficulties,

are these sufficient to determine the presence of some difficulties? – This has

been done in some research. Based on the results of this study, these reports

may occur because of undiagnosed optometric difficulties. This is an important finding and should be emphasised.

**The authors agree that questionnaire data alone is not sufficient to determine the presence of reading difficulties and that some of the questionnaire findings may be indicative of underlying optometric difficulties. This has now been stated in the discussion.**

23. At the end of page 10, the authors report that there is little interest in research in vergeance and accommodative difficulties because these can be easily treated. Evidence for this should be presented. There are many studies, see the work of Professor John Stein that have reported on the importance of binocular difficulties in children with dyslexia.

**This sentence has been changed to improve clarity and a references has been include to reflect the work of Professor Stein.**

24. Overall, this is a data rich study that should make a valuable contribution to the literature. However, I would only recommend publication after the manuscript and figures and tables have been extensively revised. Particular attention needs to be paid to the results section.

**We have addressed all the comments above and are very grateful to the reviewer for the helpful suggestions.**

Thank you for inviting me to review this interesting paper.

**Level of interest:** An article whose findings are important to those with closely

related research interests

**Quality of written English:** Acceptable

**Statistical review:** Yes, and I have assessed the statistics in my report.

**Declaration of competing interests:**

I declare that I have no competing interests.

**REVIEWER 2**

**Reviewer's report**

**Title:** A survey of visual function in an Austrian population of school-age children

with reading and writing difficulties

**Version:** 3 **Date:** 20 February 2010

**Reviewer:** Mingguang He

**Reviewer's report:**

In this study, the authors retrospectively investigated the visual function in the

children (6-14 years of age) with “reading and writing difficulties” without IQ

defect and age-matched control group in Austria. The study is carefully

conducted and interesting. The manuscript is well prepared and written. The

study demonstrates the reading difficulties in children with normal IQ could be in part explained by poor visual function, particularly binocular vision anomalies.

**The authors are pleased that this reviewer found the study well conducted and interesting. The authors are grateful to the reviewer for the helpful comments that we feel have improved the quality of the manuscript. Please find below details of how all comments have been fully addressed.**

The authors may consider to address the following concerns.

1. The control group are the children attending optometric service, will they be

likely with refractive error. How did the authors ensure they don’t have “reading and writing difficulties”.

**All ‘clinical control’ subjects have been asked whether they have reading difficulties with school work or home work. Those children or parents who report difficulties with reading have been excluded from the study and have been referred to the educational institute for an assessment (n=10). This has been clarified in the manuscript.**

2. The title of this article contains ‘writing difficulties’. However, description of

neither methodology or result of the measurement of writing difficulty was

mentioned.

**The inclusion of ‘writing difficulties’ was in response to comments from parents and children themselves regarding their difficulties in school. No children reported writing difficulties in the absences of reading difficulties and this particular wording was chosen to reflect the problems reported by the subjects. This has now been clarified in the manuscript.**

3. We understand the challenges of examining very young children particularly when lots of sophisticated tests have to be done. Should the authors report the difficulties in very young children, how reliable of the testing for the children aged 6 to 7 years of age. How the authors select the age criterion for enrolment –presumably the authors should select older children for this comparison study.

**The author (WD) is an experienced clinician with many years of practice testing young children. This has now been stated in the manuscript.**

**All tests used are standard routine procedures that are regularly employed to assess the visual status of children. Further references have been included (see list below) to demonstrate the use of the tests included in the study in childhood populations.**

[**Rutstein RP**](http://www.ncbi.nlm.nih.gov/pubmed?term="Rutstein RP"%5BAuthor%5D&itool=EntrezSystem2.PEntrez.Pubmed.Pubmed_ResultsPanel.Pubmed_RVAbstract)**,** [**Fuhr PD**](http://www.ncbi.nlm.nih.gov/pubmed?term="Fuhr PD"%5BAuthor%5D&itool=EntrezSystem2.PEntrez.Pubmed.Pubmed_ResultsPanel.Pubmed_RVAbstract)**,** [**Swiatocha J**](http://www.ncbi.nlm.nih.gov/pubmed?term="Swiatocha J"%5BAuthor%5D&itool=EntrezSystem2.PEntrez.Pubmed.Pubmed_ResultsPanel.Pubmed_RVAbstract)**.** [**Optom Vis Sci.**](javascript:AL_get(this, 'jour', 'Optom Vis Sci.');) **1993 Jun;70(6):496-500. Comparing the amplitude of accommodation determined objectively and subjectively.**

[**Montes-Micó R**](http://www.ncbi.nlm.nih.gov/pubmed?term="Montes-Micó R"%5BAuthor%5D&itool=EntrezSystem2.PEntrez.Pubmed.Pubmed_ResultsPanel.Pubmed_RVAbstract)**,** [**Ferrer-Blasco T**](http://www.ncbi.nlm.nih.gov/pubmed?term="Ferrer-Blasco T"%5BAuthor%5D&itool=EntrezSystem2.PEntrez.Pubmed.Pubmed_ResultsPanel.Pubmed_RVAbstract)**. D**[**oc Ophthalmol.**](javascript:AL_get(this, 'jour', 'Doc Ophthalmol.');) **2000 Jul;101(1):25-33. Distribution of refractive errors in Spain.**

[**Chen AH**](http://www.ncbi.nlm.nih.gov/pubmed?term="Chen AH"%5BAuthor%5D&itool=EntrezSystem2.PEntrez.Pubmed.Pubmed_ResultsPanel.Pubmed_RVAbstract)**,** [**O'Leary DJ**](http://www.ncbi.nlm.nih.gov/pubmed?term="O'Leary DJ"%5BAuthor%5D&itool=EntrezSystem2.PEntrez.Pubmed.Pubmed_ResultsPanel.Pubmed_RVAbstract)**,** [**Howell ER**](http://www.ncbi.nlm.nih.gov/pubmed?term="Howell ER"%5BAuthor%5D&itool=EntrezSystem2.PEntrez.Pubmed.Pubmed_ResultsPanel.Pubmed_RVAbstract)**.** [**Ophthalmic Physiol Opt.**](javascript:AL_get(this, 'jour', 'Ophthalmic Physiol Opt.');) **2000 May;20(3):185-98. Near visual function in young children. Part I: Near point of convergence. Part II: Amplitude of accommodation. Part III: Near heterophoria.**

4. Another potential problem in the methodology is that the observer was not

masked, or at least did not mention, to the categories of the subjects. This may bias the results and in favor of positive findings in the referred group.

**Due to the nature of the study it was not possible to ensure that the observer was masked to the subject group (referred or clinical control) however, all procedures were carried out in a standardized order using the same testing conditions for each subject. In addition, differences between control and test groups could not be discerned with any individual subject; in such a large cohort any differences only becomes apparent after statistical analysis. This point has now been addressed in the discussion.**

5. The authors reported lots of binocular function anomalies but did not describe if the anisometropia was presented differently in two groups.

**Data regarding anisometropia in both groups has now been included in the manuscript.**

6. Statistical method should be reported, i.e., for questionnaire responses.

**One way ANOVA was used and this has now been stated in the results section.**

7. Figure 1 doesn’t look good, the authors may consider use box plot. The info in some other figures may be presented in text instead of figures if the findings are not critical. Figure 7 is also problematic.

**Figures 1 and 7 have been amended and replaced with box plots. The authors agree that these graphs better represents the data. The figure legends have been amended accordingly.**

8. In the discussion, the authors may consider mention the association of phoria, binocular function and how they compromise the reading capability.

**The discussion has been amended slightly to emphasise this point.**

**Level of interest:** An article of outstanding merit and interest in its field

**Quality of written English:** Acceptable

**Statistical review:** Yes, and I have assessed the statistics in my report.

**Declaration of competing interests:**

I declare that I have no competing interests.
